# The Radioprotective Effect of Procaine and Procaine-Derived Product Gerovital H3 in Lymphocytes from Young and Aged Individuals

**DOI:** 10.1155/2020/3580934

**Published:** 2020-06-24

**Authors:** Anca Ungurianu, Denisa Margina, Claudia Borsa, Cristina Ionescu, Gudrun von Scheven, Lucie Oziol, Philippe Faure, Yves Artur, Alexander Bürkle, Daniela Gradinaru, Maria Moreno-Villanueva

**Affiliations:** ^1^Department of Biochemistry, Faculty of Pharmacy, Carol Davila University of Medicine and Pharmacy, RO-020956 Bucharest, Romania; ^2^Department of Biology of Aging, Ana Aslan National Institute of Gerontology and Geriatrics, RO-011241 Bucharest, Romania; ^3^Department of Biology, Molecular Toxicology Group, University of Konstanz, D-78457 Konstanz, Germany; ^4^Faculty of Pharmacy, CNRS UMR 8079, University of Paris-Sud, F-92296 Châtenay-Malabry, France; ^5^Centre for Taste and Feeding Behavior, UMR CNRS 6265-INRA 1324-University of Burgundy-AgroSup, F-21000 Dijon, France; ^6^Department of Sport Science, Human Performance Research Centre, University of Konstanz, D-78457 Konstanz, Germany

## Abstract

Ionizing radiation induces genomic instability in living organisms, and several studies reported an ageing-dependent radiosensitivity. Chemical compounds, such as scavengers, radioprotectors, and modifiers, contribute to reducing the radiation-associated toxicity. These compounds are often antioxidants, and therefore, in order to be effective, they must be present before or during exposure to radiation. However, not all antioxidants provide radioprotection. In this study, we investigated the effects of procaine and of a procaine-based product Gerovital H3 (GH3) on the formation of endogenous and X-ray-induced DNA strand breaks in peripheral blood mononuclear cells (PBMCs) isolated from young and elderly individuals. Interestingly, GH3 showed the strongest radioprotective effects in PBMCs from young subjects, while procaine reduced the endogenous amount of DNA strand breaks more pronounced in aged individuals. Both procaine and GH3 inhibited lipid peroxidation, but procaine was more effective in inhibiting mitochondria free radicals' generation, while GH3 showed a higher antioxidant action on macrophage-induced low-density lipoprotein oxidation. Our findings provide new insights into the mechanisms underlying the distinct effects of procaine and GH3 on DNA damage.

## 1. Introduction

Irradiation with ionizing radiation (IR) can induce mutations, cancer, and ageing in living organisms [1]. IR leads to oxidizing events causing cellular damage through direct interactions targeting macromolecules or via the free radicals produced by the radiolysis of water. These oxidizing events are amplified by endogenous cellular reactions, which further induce oxidative damage to DNA, lipids, proteins, and other molecules [2]. The basic mechanisms of radiation-induced lipid peroxidation have been previously summarized [3]. It is well known that reactive electrophilic compounds are formed during lipid peroxidation (mainly alpha and beta-unsaturated aldehydes) and can further alter DNA exhibiting both genotoxic and mutagenic actions; among them, 4-hydroxynonenal (4-HNE) is known for its genotoxic effects, while malondialdehyde (MDA) for its mutagenic ones [4]. Moreover, aldehyde-derived lipid peroxidation products can induce DNA strand breaks via oxidation of double bonds [5].

Immune cells are among the most radiosensitive cells in the body. However, their response to radiation depends on radiation type, dose, and dose rate. Both immunosuppressive and immune activating consequences were observed after high IR doses, while the effects of low doses are still controversial [6]. In fact, significant interindividual variability of radiation-related oxidative status [7] and DNA damage [8, 9] has been reported in human lymphocytes. Several cellular processes, such as defence against oxidative stress and DNA repair or telomere shortening and inflammatory pathways, may contribute to the relationship between aging and radiosensitivity [10]. Radiation contributes to the generation of reactive oxygen species (ROS), resulting in increasing amounts of cellular damage and aging. The link between radiosensitivity and aging was investigated in both healthy individuals and cancer patients, finding that the number of radiosensitive individuals increased with age in both groups [11].

Generally, antioxidants are considered to be of great interest for both radioprotection strategies [12–15] and antiageing therapies [16–18]. Radioprotective molecules may prevent the formation and facilitate the removal of free radicals, reinforce natural antioxidant systems, enhance DNA repair, reduce the postradiation inflammatory response, and even delay cellular division allowing more time for cells to undergo restorative processes or initiate apoptosis [15]. Furthermore, radioprotectors must exhibit radical-scavenging properties and antioxidant activity; however, not all antioxidants provide radioprotection [19].

Procaine was synthesized by Alfred Einhorn in 1905 and introduced in clinical practice as Novocain, becoming a local anaesthetic prototype. Procaine binds to membrane constituents and modulates a series of ion channels, interacts with membrane phospholipids, and induces changes in membrane fluidity depending on its concentration [20, 21]. Also, mitochondria, which are considered the powerhouses of the cell, are a potential target for general and local anaesthetics [22]. Procaine and its metabolites affect several biochemical and cellular processes like membrane conductance [20], oxidative phosphorylation [23], mitochondrial function and structure [24], monoamine oxidase activity [25], and DNA methylation [26]. Although Gerovital H3 (GH3) is a procaine-based preparation, its effectiveness has been disputed [27, 28]. Nevertheless, the antioxidant actions of procaine and GH3 were reported in several *in vitro* studies [29–31]. Also, it was reported that high concentrations (20 mM) of procaine inhibit DNA repair in bacteria [32].

In this context, we hypothesized that procaine might be a radioprotector, and we set out to investigate the effects of procaine and GH3 on the formation of endogenous and X-ray-induced DNA strand breaks in immune cells isolated from young versus aged individuals. Furthermore, we used specific and sensitive *in vitro* assessments to determine their efficiency in preventing lipid peroxidation of various biological samples (lymphoblastoid cells, mitochondria, human serum, and oxidized LDL).

To the best of our knowledge, there are no prior studies reporting on the effect of low doses of procaine on radiation-induced DNA damage in human cells.

## 2. Materials and Methods

### 2.1. Chemicals, Drugs, and Reagents

Procaine hydrochloride (CAS No. 51-05-8) and all other routine reagents were of the highest purity commercially available and were purchased from Sigma-Aldrich (St. Louis, MO, USA). Commercially available Gerovital H3 (GH3) (Zentiva, Romania—approved by the National Agency of Medicines and Medical Devices according to no. 1583/2012/01 governmental order) injectable solution (2% procaine hydrochloride, 0.12% benzoic acid, 0.10% potassium metabisulphite, 0.01% disodium phosphate, and pH 3.3) was purchased from a national pharmacy. To test comparatively the effect of GH3 versus the procaine effect, a working solution of 2% (*w*/*v*, in distilled water) procaine hydrochloride was prepared and systematically used in each experimental model.

### 2.2. Assessment of DNA Strand Breaks

Peripheral blood mononuclear cells (PBMCs) were obtained from the whole blood by Biocoll (Biochrome AG, Germany) density-gradient centrifugation. The venous blood was drawn from volunteers using S-Monovettes (Sarstedt, Germany). Subjects were non-smokers and healthy female or male volunteers between 24 and 77 years of age. Ethical approval was obtained from the Ethics Committee of University of Konstanz. A signed Informed Consent was obtained from each subject. Non-stimulated PBMCs (2 × 10^6^ cells/mL) from two individual groups, elderly (71 ± 6 years, *n* = 12 individuals) and young (27 ± 3 years, *n* = 12 individuals) subjects, were treated with 0, 0.25, 0.5, or 1 mM procaine or GH3 (in procaine hydrochloride equivalents) and incubated for 24 hours at 37°C in 5% CO_2_-humidified atmosphere. Thereafter, cell suspension was removed by centrifugation, and PBMCs were suspended in isotonic buffer (0.25 M mesoinositol, Sigma-Aldrich, CAS No. 87-89-8; 10 mM sodium phosphate, pH 7.4, Sigma-Aldrich, CAS No. 7601-54-9; and 1 mM magnesium chloride, Sigma-Aldrich, CAS No. 7786-30-3) and irradiated on ice with 2, 4, 6, or 8 Gy ionizing radiation (70 kV, 30 mA, 70 cm distance, 1.25 mm Al filter; Biological X-ray Irradiator X-RAD 225 iX from Precision X-ray, Inc., North Branford, USA). After irradiation, the cells were processed by a liquid handling device to assess DNA strand breaks. DNA strand breaks were quantified using the automated fluorescence-detected alkaline DNA unwinding (FADU) assay [33, 34]. Due to the automation of the technical process, the automated FADU assay accomplishes a high reproducibility and sensitivity and has been successfully applied in several studies [35–47]. This method is based on the progressive DNA unwinding (denaturation) under controlled alkaline pH, time, and temperature conditions [33, 34]. All the automated analytical steps were performed using the TECAN Genesis RSP 100-LHD equipment (TECAN AG, Hombrechtikon, Switzerland). The dye SYBR® Green (Invitrogen, Darmstadt, Germany) that specifically binds to the double-stranded DNA (nonunwound) was used. A decrease in the fluorescence intensity of SYBR® Green was measured (492 nm excitation and 520 nm emission wavelengths) indicating an increase of DNA unwinding and, hence, a rise in the number of DNA breaks. Unwinding is expressed in Gy dose equivalent as described by [48]. The effects of procaine and GH3 treatment on endogenous DNA stand breaks in nonirradiated PBMCs were also evaluated using different concentrations. Previous to the radioprotection experiments, PBMCs obtained from few young subjects were incubated with 2, 3, 5, and 10 mM of either procaine or GH3. Concentrations of procaine and GH3 of 3 mM and higher showed a genotoxic effect as measured by DNA strand breaks formation, while 2 mM of procaine did not have any protective effect or even induced DNA strand breaks (Figure S1). Therefore, 1 mM and lower concentrations of both compounds were considered for further experiments.

### 2.3. Lipid Peroxidation in Jurkat Cell Membranes

Jurkat lymphocytes (T lymphoblasts, European Collection of Cell Cultures, UK) cultured in suspension in the RPMI 1640 medium (Sigma-Aldrich, Product No. SLM-240-B) supplemented with 10% FBS (5 × 10^5^ cells/well) were incubated for 4 hours at 37°C in 5% CO_2_-humidified atmosphere, with different concentrations of procaine or GH3: 0 (control), 2.5, 5.0, and 10 mM procaine hydrochloride equivalents. Lipid peroxidation was induced by cumene hydroperoxide (CuOOH, Sigma-Aldrich, CAS No. 80-15-9). Cells treated with curcumin (Sigma-Aldrich, CAS No. 458-37-7) (0, 2.5, 5, and 10 mM, in DMSO), a known antioxidant molecule, were used as a positive control. The lipid peroxidation of the cell membrane was further monitored with the fluorescent probe DPPP (diphenyl-1-pyrenylphosphine, Thermo Fisher Scientific/Invitrogen/Molecular Probes™, Eugene, Oregon, USA) according to the procedure described by Margina et al. [49, 50]. Cell suspensions were labelled with 5 *μ*M DPPP for 20 minutes in the dark, at room temperature, and were further treated with 10 *μ*M CuOOH to induce lipid peroxidation. Fluorescence emission spectra between 360 nm and 410 nm (excitation set at 351 nm) were recorded every 2 minutes, for 18 minutes using a Perkin-Elmer LS 50B spectrofluorometer, equipped with an externally thermostatic cell holder. Maximum fluorescence intensity was measured for an emission wavelength of 380 nm as previously described. Susceptibility to lipid peroxidation of treated samples and control cells were expressed as relative fluorescence units (RFU) after time-dependent exposure to CuOOH.

### 2.4. Lipid Peroxidation in Rat Liver Mitochondria

Amplex Red (N-acetyl-3,7-dihydroxyphenoxazine, Life Technologies, Grand Island, NY, USA) proved to be a versatile probe which can be applied for the assessment of fatty acid hydroperoxides, H_2_O_2_, and other ROS in isolated mitochondria [51–53]. Crude liver mitochondrial fraction was obtained from a 3-month-old Wistar rat by differential centrifugation, using the protocol described by Wieckowski et al. [54]. The study was approved by the local Animal Ethics Committee at Carol Davila University of Medicine and Pharmacy, and all procedures were carried out according to the Directive 86/609 EEC guidelines for the care and use of laboratory animals. Mitochondrial preparations (standardized for a protein content of 0.3 mg protein/mL phosphate buffer 20 mM, pH 7.3) were incubated for 10 minutes at room temperature (25°C) in a 1 : 1 ratio with sodium succinate (0.05 M, Sigma-Aldrich, CAS No. 150-90-3). Different procaine and GH3 volumes were added to reaction mixture, corresponding to 0 (control), 0.5, 1.0, 2.0, 5.0, and 10 mM procaine hydrochloride final concentrations in the samples. After the addition of Amplex Red working solution (75 *μ*M), the samples were incubated for 10 minutes at room temperature and protected from light. The mixture was diluted to 2000 *μ*L with PBS 1x before reading at 585 nm emission wavelength after excitation to 544 nm on a LS 50B Fluorescence Spectrometer from Perkin-Elmer. The measurements were conducted versus a control mitochondrial sample without procaine or GH3. Also, for each experiment, a blank was prepared, containing Amplex Red working solution plus PBS 1x, in order to assess the probe's autofluorescence. Results were expressed as lipid peroxidation inhibition in percentage (%), calculated from the recorded fluorescence as relative fluorescence units (RFU), using the following equation: [(RFU_Control_ − RFU_Samples_)/RFU_Control_] × 100.

### 2.5. Lipid Peroxidation in Human Serum

Serum samples were obtained from four healthy adult volunteers—students from the Faculty of Pharmacy, Carol Davila University of Medicine and Pharmacy. Ethical approval for collecting the peripheral venous blood from human subjects was obtained from the Carol Davila University of Medicine and Pharmacy Ethics Committee. Serum lipoprotein concentrates were isolated from each serum sample following centrifugation at 7000 x *g*, 4°C, 60 min, on Amicon Ultra-10 k Centrifugal Filter Units 10.000 NMWL (Millipore). The assessment of lipid hydroperoxides in serum lipoprotein concentrates using the Amplex Red fluorescent probe was performed according to the method previously described [52, 53, 55].

Briefly, 50 *μ*L of serum concentrate samples (standardized for a protein content of 0.5 mg protein/mL phosphate buffer 20 mM, pH 7.3) was incubated for 10 minutes at room temperature (25°C) with different procaine and GH3 concentrations: 0 (control), 0.5, 1.0, 2.0, 5.0, and 10 mM. After the addition of 50 *μ*L Amplex Red working solution (300 *μ*M), the samples were incubated for 10 minutes at room temperature and protected from light. The samples were diluted to 2000 *μ*L with PBS 1x before the fluorescence measurements as above described. Results were also expressed as lipid peroxidation inhibition in percentage (%).

### 2.6. Macrophage-Induced LDL Oxidation

The native LDL fraction was isolated from human fresh plasma using the density-gradient ultracentrifugation and prepared as described previously [56, 57]. Human plasma was obtained from normolipidemic, apparently healthy volunteers selected by the Blood Donation Service of Burgundy/Franche-Comté, France. The human U937 monocyte-like cell line (European Collection of Cell Cultures, UK) was grown in the RPMI-1640 culture medium (Sigma-Aldrich, Product No. SLM-240-B) enriched with 10% heat-inactivated fetal calf serum and incubated for 48 hours with phorbol 12-myristate 13-acetate (Sigma-Aldrich, CAS No. 16561-29-8) for cell activation and differentiation into macrophages [58]. The adherent macrophages (0.5 × 10^6^ cells/mL, in 6-well plates) were further used for LDL oxidation experiments. The growth medium was replaced with the oxidation mixture containing serum-free Ham's Nutrient Mixture F-12 (Sigma-Aldrich, CAS No. 51651C) without phenol red, 8 *μ*M FeSO_4_ (Sigma-Aldrich, CAS No. 7782-63-0), and pure LDL at a final concentration of 100 *μ*g protein/mL [58]. The cells were exposed for 24 hours at 37°C in a 5% CO_2_-humidified atmosphere to the oxidation medium supplemented in different procaine and GH3 concentrations: 0 (control), 0.5, 1.0, and 2.0 mM procaine hydrochloride equivalents. Lipid peroxidation end-products generated by macrophages were estimated by thiobarbituric acid reactive substances (TBARS) measurements in the cell culture medium. After incubation, the cell oxidation medium was removed, and lipid peroxidation was stopped with 1 mM EDTA (Sigma-Aldrich, CAS No. 60-00-4) and 0.2 mM butylhydroxytoluene (BHT, Sigma-Aldrich, CAS No. 25013-16-5) at 4°C. Detached cells present in the medium mixture were discarded by centrifugation (207 x *g*, 5 min). The TBARS from the supernatant were determined according to [58] with trichloracetic (TCA, Sigma-Aldrich, CAS No. 76-03-9)/thiobarbituric (TBA, Sigma-Aldrich, CAS No. 504-17-6)/hydrochloric (HCl, Sigma-Aldrich, CAS No. 7647-01-0) acid (13.6/0.36/2.4%, *w*/*v*) reagent mixture and measured at 532 nm, using a Lambda Bio10 Perkin-Elmer spectrophotometer. TBARS were expressed as nmoles of malondialdehyde (MDA) using a calibration curve of 1,1,3,3-tetramethoxypropane (Sigma-Aldrich, CAS No. 102-52-3). Results were expressed as lipid peroxidation inhibition in percentage (%), calculated using the following equation: [(TBARS_Control_ − TBARS_Samples_)/TBARS_Control_] × 100.

## 3. Results

### 3.1. Protective Effects of Procaine and GH3 on DNA Strand Breaks in Young versus Aged Individuals

**Figure 1:**
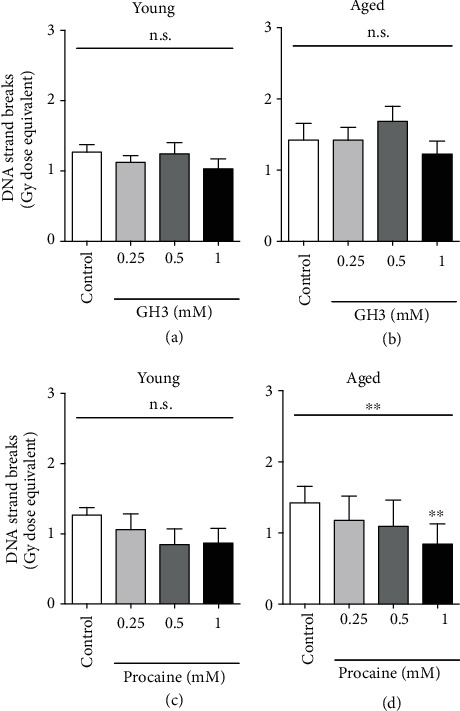
*In vitro* inhibitory effect of procaine (c), (d) and GH3 (a), (b) on DNA strand breaks in nonirradiated peripheral blood mononuclear cells (PBMCs) isolated from young (27 ± 3 years) and aged (71 ± 6 years) individuals. Cells from young and aged subjects were divided in GH3- or procaine-treated or nontreated (control) groups, respectively. The cells were treated for 24 hours with 0, 0.25, 0.5, or 1 mM of procaine hydrochloride equivalents. Statistical analyses were performed using Friedman's test and Dunn's multiple comparisons test. Values, in Gy dose equivalent, represent means and SEM of 12 different individuals in each group. ^∗^Statistical significance when comparing against nontreated (control) samples, ^∗∗^*p* < 0.01; n.s.: nonsignificant.

DNA damage is known to be one of the mechanisms responsible for increasing mutagenesis risk, being involved in cellular survival, vascular aging, cancer, and neurodegenerative diseases [38, 59]. We hypothesized that procaine and GH3 protective effects would be more pronounced in older compared to young subjects. Treatment with GH3 (containing the same concentration of procaine) (Figures 1(a) and 1(b)) did not lead to any significant change in the amount of endogenous DNA strand breaks either in young or in aged individuals (two-way ANOVA *p* > 0.05). Contrary, treatment with different procaine concentrations significantly reduced the amount of endogenous DNA strand breaks in a dose-dependent manner (two-way ANOVA *p* = 0.0002) but independent of age. However, treatment with 1 mM procaine decreased the amount of endogenous DNA strand breaks in aged (Friedman's *p* = 0.0049; Dunn's multiple comparisons test *p* = 0.0015) but not in young individuals (Friedman's *p* = 0.1116) when compared to their own group nontreated controls (Figures 1(c) and 1(d)).

**Figure 2:**
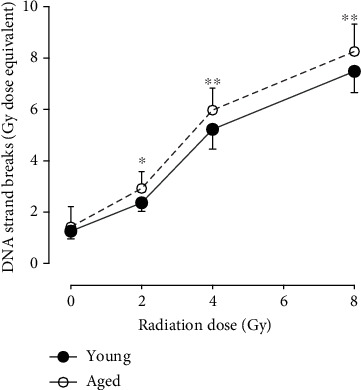
Endogenous and *ex vivo* radiation-induced DNA strand breaks in PBMCs isolated from young (27 ± 3 years, black circles) versus aged (71 ± 6 years, white circles) individuals. Lines represent results from nonirradiated (radiation dose = 0 Gy) and irradiated cells (radiation dose = 2, 4, or 8 Gy). Statistical analyses were performed using two-way RM ANOVA and Sidak's multiple comparisons test. Values represent means and standard deviations of 12 individuals in each group. ^∗^Statistical significance compared to young individuals, ^∗^*p* = 0.0366; ^∗∗^*p* < 0.01.

Reduced antioxidant capacity [1, 60] and decreased DNA repair [61–63] are associated with ageing. Thus, it is conceivable that IR may induce higher DNA damage in PBMCs from elderly individuals than in those from young people. As expected, there was a significant (two-way ANOVA RM age × dose interaction *p* = 0.0092) effect of age on radiation-induced DNA strand breaks (Figure 2). All radiation doses (2, 4, and 8 Gy) induced a significantly higher number of DNA strand breaks in cells from aged individuals when compared with young individuals.

**Figure 3:**
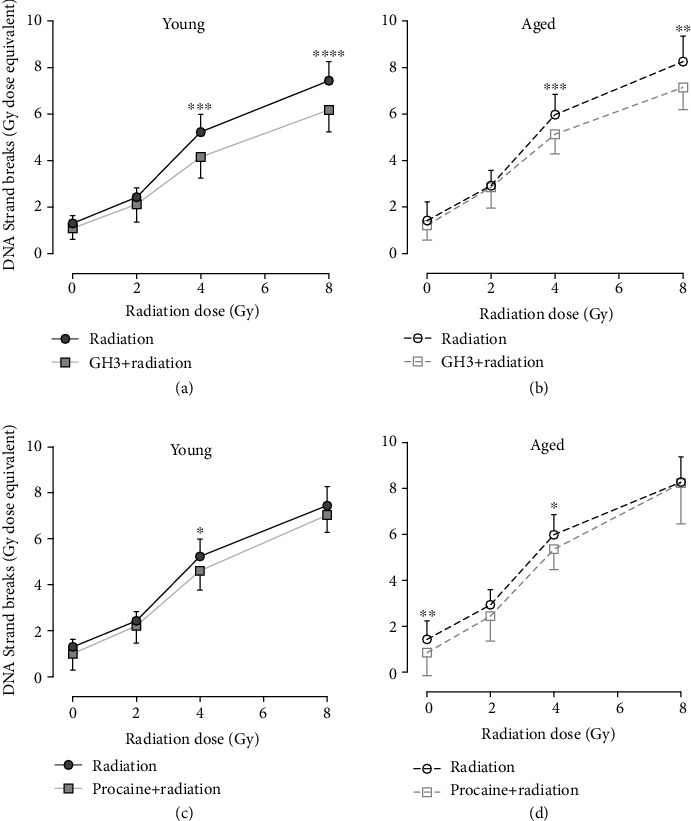
*In vitro* inhibitory effect of GH3 (1 mM) (a), (b) and procaine (1 mM) (c), (d) on DNA strand break formation in irradiated peripheral blood mononuclear cells (PBMCs) isolated from young (27 ± 3 years) and aged (71 ± 6 years) individuals. Cells were irradiated with 0, 2, 4, or 8 Gy 24 hours after treatment with 1 mM of procaine hydrochloride equivalents. Statistical analyses were performed using two-way RM ANOVA and Sidak's multiple comparisons test. Values represent means and standard deviations of 12 different individuals in each group. Statistical significance when comparing against nontreated (radiation) samples, ^∗^*p* < 0.05; ^∗∗^*p* = 0.0085; ^∗∗∗^*p* < 0.0008; ^∗∗∗∗^*p* < 0.0001.

Pretreatment with GH3 before radiation significantly affected the radiation dose response (significant interaction between radiation and GH3 treatment; *p* < 0.0005) in both young and aged individuals (Figures 3(a) and 3(b)). This effect was significant at radiation doses of 4 and 8 Gy (*p* < 0.005). Contrary, procaine (Figures 3(c) and 3(d)) did not affect the radiation dose response (no significant interaction between radiation and procaine treatment; *p* > 0.5) in either young or aged subjects.

### 3.2. Protective Effects of Procaine and GH3 against Lipid Peroxidation

In order to elucidate the distinct effects of procaine and GH3 on DNA damage, we evaluated the efficiency of different procaine and GH3 concentrations in preventing lipid peroxidation in various *in vitro* experimental models.

**Figure 4:**
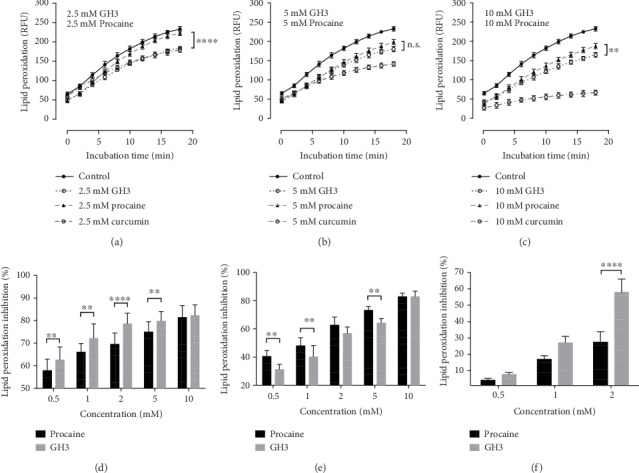
*In vitro* inhibitory effect of procaine and Gerovital H3 (GH3) on lipid peroxidation in Jurkat cells (a–c), human serum (d), rat liver mitochondria (e), and human macrophage-induced LDL oxidation (f). Jurkat cells were preincubated with 0 (control), 2.5 (a), 5.0 (b), or 10 mM (c) of procaine hydrochloride equivalents for the indicated time points. Lipid peroxidation was induced by cumene hydroperoxide, and curcumin served as a control for inhibitory effect on lipid peroxidation. Human serum (d) and rat liver mitochondria (e) samples were pretreated with 0 (control), 0.5, 1.0, 2.0, 5.0, or 10 mM of procaine hydrochloride equivalents. Human macrophage-induced LDL oxidation (f) was assessed in human U937 monocyte-like cell line. Cells were treated with 0 (control), 0.5, 1.0, or 2.0 mM of procaine hydrochloride equivalents, and TBARS was quantified. Statistical analyses for (a–c) and (f) were performed using two-way ordinary ANOVA and Sidak's multiple comparisons test. Statistical analyses for (d, e) were performed using two-way RM ANOVA and Sidak's multiple comparisons test. Error bars mean standard deviations of 3 or 4 experiments. Statistical significance between GH3 and procaine: ^∗∗^*p* < 0.01; ^∗∗∗∗^*p* < 0.0001.

First of all, the antioxidant effects of procaine and GH3 were assessed in a human lymphoblastoid cell line. The purpose of this experiment was to evaluate the effects of procaine and GH3 using a cellular experimental model that mimics physiological targets to be protected *in vivo* against oxidative stress resulting from proinflammatory conditions (Figure 4). Procaine and GH3 similarly reduced the generation of cell membrane lipoperoxides at 5 and 10 mM concentrations (Figures 4(b) and 4(c)). However, at the lowest concentration (2.5 mM), their effects significantly differed (*p* < 0.0001), as GH3 was more effective in reducing the generation of membrane lipoperoxides, showing similar activity to curcumin (Figure 4(a)).

Both procaine and GH3 showed a significant (*p* < 0.0001) dose-dependent inhibitory effect on lipid peroxidation in human serum samples and in rat liver mitochondria. Interestedly, in human serum (Figure 4(d)), GH3 showed a significantly (*p* < 0.0001) higher lipid peroxidation inhibition compared to procaine, whereas in rat liver mitochondria (Figure 4(e)), the inhibitory effect of procaine was significantly higher (*p* < 0.0001).

Further, the effect of procaine and GH3 on cell-mediated induced LDL oxidation was investigated. In this purpose, LDL lipoperoxides were generated by incubating macrophages differentiated from human monocytic U973 cells with human native LDL under prooxidant conditions. End-products of lipid peroxidation such as malondialdehyde (MDA) and 4-hydroxynonenal (HNE) were measured as thiobarbituric acid reactive substances (TBARS). At all tested concentrations of procaine and GH3 significantly (*p* < 0.0001) inhibited TBARS formation. However, the inhibitory effect of GH3 was significantly (*p* < 0.0001) higher than that of procaine for the 2 mM concentration (Figure 4(f)).

## 4. Discussion

Ionizing radiation (IR) induces DNA damage and increases the risk of cancer [64]. Furthermore, the risk of radiation-induced carcinogenesis rises with age [10]. Therefore, taking age into consideration when investigating the effects of antioxidants, radioprotectors and scavengers on radiation-induced DNA damage, is of great interest in environmental, occupational, and medical research.

A significant fraction of DNA damage produced by IR is caused by free radicals generated during water radiolysis [65]. Since the repair capacity of ROS-induced DNA damage was reported to decrease with age [66], it is reasonable to hypothesize that radiation induces more cellular damage in older individuals. As mentioned before, radioprotective molecules may not only prevent the formation and facilitate the removal of free radicals but also reinforce natural antioxidant systems and enhance DNA repair. In order to allow cells to adjust their biochemistry in response to procaine and GH3, PBMCs were incubated for 24 hours. Furthermore, in order to avoid confounding effects due to immune stimulation through mitogens, we investigated the effect of procaine and GH3 in quiescent PBMCs known to be in G0 phase of the cell cycle [67]. Our results show that cells from older subjects were more susceptible to radiation. Whether or not these findings are in accordance with previously published data is difficult to assess. Early studies reported that the survival rate of lymphocytes from elderly individuals after *ex vivo* irradiation is approximately one-half from that of lymphocytes from young individuals [68]. Discordantly, a more recent study found an age-associated decrease in IR-induced apoptosis [69], and this finding was also confirmed in later studies [70, 71]. Regarding radiation-induced DNA strand breaks, the number of *γ*-H2AX foci is higher in young than in old mice, which partially correlated with cellular proliferation and expression of DNA repair proteins [72]. However, in a human study including 172 individuals between 40 and 77 years of age, *ex vivo* irradiation of lymphocytes showed no significant differences in the induction of DNA strand breaks in aged versus young individuals [38]. Contrary, in another study including 31 individuals between 25 and 91 years old, an age-dependent increase in DNA single-strand breaks was observed in human lymphocytes immediately after *ex vivo* irradiation [73]. These discrepancies could be explained by the different experimental designs, cohorts, and/or methodologies. Further research is necessary in order to identify the factors that induce age-dependent radiosensitivity.

In this study, we did not detect any significant differences regarding the number of endogenous DNA strand breaks (without radiation) between young and elderly individuals. Our results showed that treatment of PBMCs with GH3 reduced radiation-induced DNA strand breaks in both groups. However, it should be noted that the protective effect was slightly higher in young subjects, which could be explained by a higher interindividual variability in the aged group. Furthermore, the endogenous DNA strand breaks observed in nonirradiated cells could be reduced by procaine. There was a significant protective effect of 1 mM procaine only within the elderly group probably due to the observed tendency of accumulating DNA strand breaks in aged individuals, but age did not have a significant effect on procaine dose response. Therefore, whether or not age influences the protective effect of procaine needs to be further investigated. These findings are intriguing: although the main active compound in GH3 is procaine, GH3 showed a protective effect against radiation, while procaine reduced the endogenous level of DNA strand breaks suggesting a slightly different mode of action.

Consequently, we aimed to evaluate whether procaine and GH3 differ in their antioxidant properties. In order to corroborate their antioxidant effects, we used novel, sensitive and specific, fluorescent *in vitro* methods, as well as various biological samples, relevant targets of oxidative damage such as lymphoblastoid cells, mitochondria, human serum, and oxidized LDL.

In our experiments comprising Jurkat cells, the lowest GH3 concentration showed similar effects to those induced by curcumin, while higher concentrations of procaine were needed to reach similar effects. Curcumin was used as a positive control of inhibition of lipid peroxidation, due to its well-known antioxidant action [50]. These results indicate that GH3 might have a higher capacity of preventing lipid peroxidation in a cellular system. Furthermore, in serum- and mitochondria-based assays, the antioxidant capacity of procaine and GH3 increased in a dose-dependent manner. However, differences between the two compounds were also observed. While procaine was slightly more effective in protecting the mitochondrial membrane, GH3 was more efficient against serum lipoperoxidation. These outcomes could be explained through the different lipid or lipoprotein microenvironments present in these biological systems and/or through the different intrinsic antioxidant capacities or ROS scavenging actions of procaine and GH3, in counteracting or preventing lipid peroxidation [29–31]. Moreover, the reduced number of endogenous DNA strand breaks observed at 24 hours postexposure to procaine could be explained through inhibition of mitochondrial ROS production. Mitochondrial ROS also increases with age [74], which could explain the higher inhibition in aged individuals. In our macrophage-induced LDL oxidation model, GH3 showed a higher antioxidant effect than procaine, similar to the previously presented results for serum lipoproteins. These observations could provide additional information about the potential effect of procaine and GH3 regarding the oxLDL-macrophage interaction in the endothelial microenvironment [75].

Globally, we highlight the dose-dependent antioxidant effect of procaine and GH3; whereupon, GH3 seems to have a higher antioxidant capacity in serum and lipid- and lipoprotein-enriched biological samples compared to procaine. One could argue that GH3 also contains additional ingredients (potassium salts, benzoic acid—used as stabilizers and preservatives in the formulation of procaine hydrochloride) which could act as antioxidants. Indeed, in previous studies, using a nonenzymatic *in vitro* system for superoxide (O_2_^.-^) generation, the antioxidant action of GH3 was attributed also to some GH3 of its components, [31, 76, 77]. However, for DNA damage experiments, cells were incubated 24 hours and thereafter centrifuged and suspended in isotonic buffer prior to radiation. Therefore, the GH3-mediated inhibition of radiation-induced DNA strand breaks cannot be explained by the presence of scavengers or antioxidants in cell culture medium; more likely, it is a matter of intracellular mechanisms, which could be attributed to procaine rather than to other additives in GH3. Furthermore, due to the higher procaine stability in GH3, the scavenger properties of procaine could be maintained for longer periods of time, explaining the GH3 protective effect on radiation-induced DNA strand breaks, in this case for both young and elderly individuals.

## 5. Conclusion

Due to the absence of recent and rigorous experimental studies involving procaine and GH3, we considered useful to explore the effects of procaine versus GH3 in the study of DNA damage and lipid peroxidation. In this work, we provide new evidence for differences in the antioxidant properties of procaine and GH3. Furthermore, procaine seems to affect the endogenous DNA strand breaks formation while GH3 prevents the radiation-induced DNA strand breaks. Age did not have any effect on GH3 treatment while procaine seems to have a slightly higher effect in the aged group although this remains to be confirmed by future extended and controlled studies. We conclude that both compounds, procaine, and GH3 have a different effect on DNA strand breaks formation which could be explained by their different antioxidative impact. Our findings are novel and constitute the basis for future studies.

## Data Availability

The data used to support the findings of this study are available from the corresponding author upon request.

## Supplementary Materials

Supplementary Materials Figure S1: effect of different procaine and GH3 concentrations on endogenous DNA strand break formation in PBMCs from three or two young subjects. Lines indicate an increase or decrease in the amount of DNA strand breaks after 24 h of *ex vivo* incubation when compared to their own initial (“physiological”) DNA strand breaks amount in each subject.
