# Supplementary material for: The Radioprotective Effect of Procaine and Procaine-Derived Product Gerovital H3 in Lymphocytes from Young and Aged Individuals
Source: Oxid Med Cell Longev. 2020 Jun 24;2020:3580934. doi: 10.1155/2020/3580934 (PMC7334788; doi:10.1155/2020/3580934)
Supplement: Supplementary Materials — Figure S1: effect of different procaine and GH3 concentrations on endogenous DNA strand break formation in PBMCs from three or two young subjects. Lines indicate an increase or decrease in the amount of DNA strand breaks after 24 h of ex vivo incubation when compared to their own initial (“physiological”) DNA strand breaks amount in each subject. [file 3580934.f1.pdf]

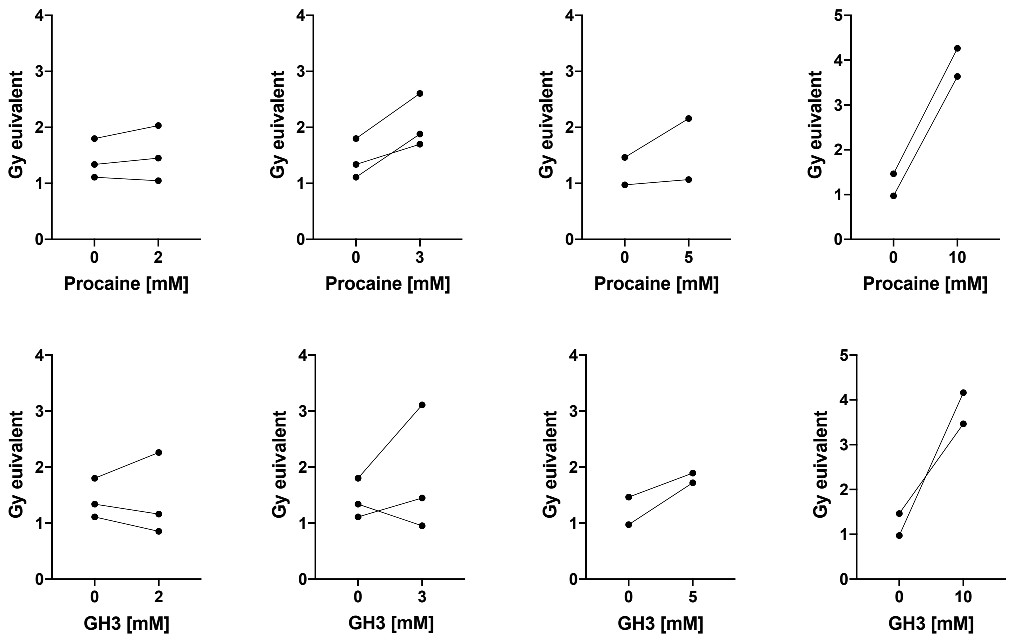

Supplementary figure 1: Effect of different procaine and GH3 concentrations on endogenous DNA strand breaks formation in PBMCs from three or two young subjects. Lines indicate an increase or decrease in the amount of DNA strand breaks after 24h of *ex vivo* incubation when compared to their own initial ("physiological") DNA strand breaks amount in each subject.
